# A cut-off value for nuchal translucency in the prediction of composite adverse outcome in cystic hygroma cases

**DOI:** 10.1590/1806-9282.20241339

**Published:** 2025-06-16

**Authors:** Murat Haksever, Atakan Tanacan, Hakki Serbetcı, Osman Onur Ozkavak, Refaettin Sahın, Ekin Ersoy, Ozgur Kara, Dilek Sahin

**Affiliations:** 1 Turkish Ministry of Health, Ankara City Hospital, Department of Obstetrics and Gynecology, Division of Perinatology – Ankara, Turkey.; 2 University of Health Sciences, Turkish Ministry of Health, Ankara City Hospital, Department of Obstetrics and Gynecology, Division of Perinatology – Ankara, Turkey.

**Keywords:** Fetal cystic hygroma, Nuchal translucency, Pregnancy

## Abstract

**OBJECTIVE:**

The aim of this study was to determine a cut-off value for nuchal translucency in the prediction of composite adverse outcome in cystic hygroma cases.

**METHODS:**

This study was designed retrospectively in the perinatology department of a tertiary hospital. All fetuses followed up with a diagnosis of cystic hygroma between 2019 and 2023 were included in the study. Demographic characteristics, prenatal ultrasound findings, non-invasive screening test results, invasive diagnostic test results, clinical management, and postnatal outcomes were evaluated. A total of 34 patients diagnosed with cystic hygroma by prenatal ultrasonography were included in the study. The 50th percentile nuchal translucency value of cystic hygroma cases was 5.2 mm. Cystic hygroma cases were divided into two groups as nuchal translucency <5.2 mm and ≥5.2 mm. A receiver operator characteristics analysis was performed to determine an optimal cut-off value of nuchal translucency in the prediction of composite adverse outcome.

**RESULTS:**

There were no statistically significant differences between the groups for chromosomal/structural abnormalities and fetal losses. Chorionic villus sampling was performed in 28 patients, and amniocentesis was performed in one patient; five patients did not accept any invasive procedures. Nineteen cases had a chromosomal abnormality in chorionic villus sampling, while nine had a normal karyotype. The result of the amniocentesis was trisomy 18. A cut-off value of 4.03 mm was found for nuchal translucency in the prediction of composite adverse outcome (76.3% sensitivity, 66.7% specificity).

**CONCLUSIONS:**

Composite adverse outcome seems to be more common in cystic hygroma cases with an nuchal translucency ≥4.03 mm.

## INTRODUCTION

Cystic hygroma (CH) is a congenital anomaly of the lymphatic system with an incidence of 1 in 6,000^1^. CH is characterized by the presence of edema and fluid-filled spaces in the posterior neck and back of a fetus at sites where the lymphatic and venous systems connect. It can occur as an isolated finding or be associated with other abnormalities as part of a syndrome^2^. Environmental, genetic, or unknown factors can cause CH. Chromosomal abnormalities are present in approximately 50% of cases^3^. Chromosomal or structural anomalies are associated with a poor prognosis. In the first or early second trimester, this condition can be diagnosed by ultrasound, with the measurement of fetal nuchal translucency (NT) in the mid-sagittal plane. If CH is diagnosed, the fetus should undergo a thorough anatomical screening for other systemic abnormalities. Subsequently, invasive fetal karyotyping should be offered to parents to detect any chromosomal abnormalities^4^. Should the proposal be accepted, an obstetrician will perform chorionic villus sampling (CVS) or amniocentesis (AS) to facilitate further diagnosis. Previous research has indicated that pregnancies involving fetal CH and associated aneuploidy have resulted in worse perinatal outcomes. However, our knowledge is limited regarding the impact of NT value on the adverse outcomes in CH cases. For this reason, the main objective of the present study is to determine a cut-off value of NT in predicting composite adverse outcome in fetuses diagnosed with CH.

## METHODS

This study was designed retrospectively in patients followed up for CH in the perinatology clinic of Ankara Bilkent City Hospital between January 2020 and August 2023. The study protocol was approved by the institutional ethics committee with ­reference number E2-23-5193, and written informed consent was obtained from all participants. Demographic characteristics, prenatal ultrasound findings, noninvasive screening test results (combined test), invasive diagnostic test results, accompanying congenital anomalies, and postnatal outcomes in fetuses that reached delivery were evaluated. Combined tests including NT, pregnancy-associated plasma protein A (PAPP-A), and beta-human chorionic gonadotropin (β-hCG), were used as the aneuploidy screening test for all cases. The risk assessment for aneuploidy was performed based on the combined test results. Apgar scores and neonatal intensive care admissions were assessed for babies born alive. All cases were evaluated by the same expert perinatologist (O.K.) using a Voluson E10 with a 2–9 MHz abdominal convex probe. The data were statistically analyzed using Statistical Package for the Social Sciences SPSS 22 (IBM Corp., NY).

The 50th percentile of NT was found to be 5.2 mm in the study population, and the cases were divided into two groups as NT<5.2 mm and ≥5.2 mm. Demographic features, clinical outcomes, fetal complications, and chromosomal ­abnormality rates were compared between the groups. Furthermore, a composite adverse outcome, which was defined as the presence of at least one adverse outcome like intrauterine fetal demise, chromosomal abnormality, hydrops fetalis, or fetal structural abnormality, was compared between the groups. A receiver operator characteristics (ROC) analysis was performed to determine the optimal cut-off value of NT in the prediction of composite adverse outcome.

The Kolmogorov-Smirnov test was applied to test whether data distribution was normal. Means and standard deviations were used for values that were applied to continuous variables that had a normal distribution. Whereas, median and range values were utilized to display continuous variables with non-normal distribution. Categorical variables were presented as numbers and percentages. As the data were not normally distributed, the Mann-Whitney U test was conducted to compare median values between the groups. ROC analysis and the Youden index were performed to determine optimal cut-off values of NT in the prediction of composite adverse outcome. A p<0.05 was regarded as statistically significant.

## RESULTS

Thirty-four patients diagnosed with CH by prenatal ultrasonography were included in the study. The 50th percentile of NT value for CH cases was 5.2 mm. CH cases were divided into two groups as equal or above and below this value. The two groups were similar in terms of maternal age, gravidity, parity, gestational age at diagnosis, maternal age over 35 years, fetal gender, fetal hydrops, fetal cardiac anomaly, fetal chromosomal anomaly, and intrauterine exitus. Composite adverse outcome was statistically higher in NT≥5.2 mm group [n=15(75%) vs. n=23(95.8%), p=0.045], respectively (Table 1).

**Table 1:** Comparison of demographic features, clinical characteristics, and perinatal outcomes of cystic hygroma cases between nuchal translucency <5.2 mm and nuchal translucency ≥5.2 mm.

	NT<5.2 mm	NT≥5.2 mm	p-value
Age	33 (8)	29 (7)	0.14
Gravidity	3 (2)	2 (2)	0.087
Parity	2 (1)	1 (2)	0.34
Gestational age at diagnosis	12 (2)	13 (1)	0.093
Maternal age over 35	6 (35.3%)	2 (11.8%)	0.106
Fetus gender (male)	9 (52.9%)	4 (23.5%)	0.08
Fetal hydrops	5 (29.4%)	4 (23.5%)	0.69
Fetal cardiac anomaly	3 (18.8%)	5 (29.4%)	0.47
Intrauterine exitus	5 (25%)	9 (37.5%)	0.37
Fetal chromosomal anomaly	11 (64.7%)^a^	14 (82.4%)^b^	0.094
Composite adverse outcome	15 (75%)	23 (95.8%)	0.045

NT: nuchal translucency, p-values less than 0.05 accepted as statistically significant.

a There were four cases with trisomy 18, five cases with trisomy 21, and two cases with monosomy X in the NT<5.2 mm group.

b There were two cases with trisomy 18, six cases with trisomy 21, and six cases with monosomy X in the NT>5.2 mm group.

The risk of chromosomal abnormality was found to be high in 16, intermediate in 10, and low in 8 patients, respectively. Thirteen fetuses were male and 21 were female. CVS was performed in 28 patients, and AS was performed in one patient. Five patients did not accept invasive procedures. CVS was performed in 28 patients. Nine were found to have a normal karyotype among the CVS cases. Seven of these patients had trisomy 21, eight had Turner syndrome, and four had trisomy 18. One case of trisomy 18 was identified through AS. Fourteen patients were terminated due to chromosomal abnormalities, including trisomy 18 (n=4), trisomy 21 (n=4), and Turner syndrome (n=4).

One patient with a normal karyotype result was terminated due to preterm premature rupture of membranes. One patient without a karyotype analysis was terminated due to a major congenital anomaly. Of the remaining 20 patients, 5 resulted in missed abortion before the 20th week of gestation, 5 resulted in intrauterine death, and 10 pregnancies were ­delivered. Of the five fetuses that resulted in miscarriage, two were diagnosed with Turner syndrome, one with trisomy 21, and one with trisomy 18. One fetus was not diagnosed because the parents did not give consent to invasive procedures. Of the five fetuses with intrauterine death, three had a normal karyotype, one had Turner syndrome, and one could not be karyotyped. Eight of the live newborns were admitted to the neonatal intensive care unit due to pleural effusion (n=1), cardiac anomalies (n=1), omphalocele (n=1), skeletal dysplasia (n=1), hypoplastic left heart syndrome (n=1), respiratory distress syndrome (n=1), and preterm birth (n=2). Cardiac abnormalities were identified in 8 of 34 fetuses diagnosed with CH. The prevalence of cardiac anomalies was higher in fetuses with Turner syndrome. Diffuse hydrops was observed in 9 of 34 patients. Six patients had no chromosomal or structural abnormalities, nor fetal loss.

**Figure 1:**
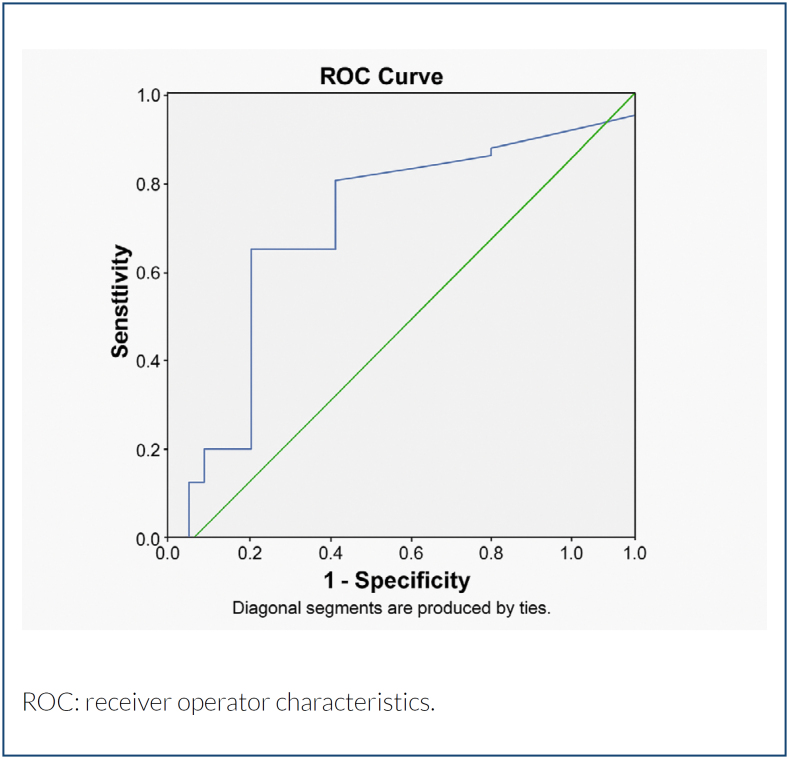
Receiver operator characteristics curve of optimal cut-off value of nuchal translucency in the prediction of composite adverse outcome in the cystic hygroma group.

A summary of ROC analysis demonstrating the optimal cut-off value of NT in the prediction of composite adverse outcome in the CH group is shown in Table 2. The optimal cut-off value was 4.03 (76.3% sensitivity, 66.7% specificity), respectively (Figure 1).

**Table 2:** Summary of receiver operator characteristics analysis demonstrating the optimal cut-off values of nuchal translucency in the prediction of composite adverse outcome in the cystic hygroma group.

Variable	Cut-off	AUC	Sensitivity	Specificity	95%CI	p
NT	4.05	0.69	76.3%	66.7%	0.44–0.94	0.13

AUC: area under the curve; CI: confidence

ROC: receiver operator characteristics.

## DISCUSSION

The results of the present study revealed that although the rates of chromosomal/structural abnormality and fetal loss were similar between the groups, the overall composite adverse outcome was higher in cases with larger NT values. Moreover, an optimal NT cut-off value could be determined in predicting composite adverse outcome. Although, the rates of chromosomal abnormality, intrauterine fetal demise, and fetal structural abnormality were comparable between the groups. The composite adverse outcome rate, defined as the presence of at least one of the complications, was higher in the greater NT group. The main goal of maternal–fetal medicine is to provide a healthy neonate to the parents. Thus, combining all complications in a group represents the rate of adverse perinatal outcomes more objectively.

In a retrospective study conducted with 193 patients with NT≥6.5 mm. Invasive procedures were performed in 115 (59.3%) cases. Fetal chromosomal results were abnormal in 81 (70.4%) cases, including 58.0% of Turner syndrome, 21.0% of trisomy 18, 11.1% of trisomy 21, and 3.7% of trisomy 13. In this study, septations were observed in all cases. Septation was identified as a unique risk factor separate from NT thickness. It appeared that the existence of septation should not be a major consideration in prenatal counseling. The study findings indicated that a significantly large NT poses a high risk of chromosomal abnormality, with no notable increase in risk based on the presence of septation or ultrasound malformations. The fetal structural abnormalities presented in the mentioned study were congenital heart defect, hydrops fetalis, omphalocele, and skeletal dysplasia^5^. Similar to the mentioned study, composite adverse outcome was more common in cases with larger NT values in our study. However, our study did not investigate the impact of septation.

Over a span of 10 years, a total of 944 fetuses with nuchal CH diagnosed through first-trimester ultrasound were included in a retrospective cohort study. Karyotype results were available for 729 fetuses, of which 329 (45.1%) were normal and 400 (54.9%, 95%CI 51–58%) were abnormal. The NT thickness showed a significant increase in 298 fetuses with an abnormal karyotype, ranging from 1.4 to 24.7 mm, and a median of 6 mm (interquartile range 2–16 mm). In comparison, 242 fetuses with a normal karyotype had a range of 1.6 to 14.4 mm, with a median of 4.1 mm (interquartile range 1.9–12 mm, p<0.001). The authors also reported that every 1 mm increase in the NT thickness was associated with higher rates of adverse outcomes like abnormal karyotype, major structural anomalies, and perinatal loss. The difference in median NT was found to be statistically significant based on karyotype (p<0.001). In both univariable and multivariable regression analyses examining the correlation between NT thickness and outcomes, higher NT thickness was linked to abnormal ­karyotype, major congenital anomaly, perinatal loss, and adverse outcome^6,7^. The results of this study were consistent with the findings of the present study.

In a retrospective study including 64 patients, prenatal and postnatal findings were investigated. CH without septa was found in 39 (60.9%) and with septa in 25 (39.1%) patients. Chromosomal abnormalities were detected in 25 (39%) patients. The most common chromosomal abnormality in CH without septa was trisomy 21 (27.8%). However, the most common chromosomal defect in CH without septa was Turner syndrome (23.8%). Survival was worse in those with additional structural abnormalities. Nine (14%) of the infants were born alive and were followed up postnatally^8^.

In one study, 29 cases of CH were followed up in two centers over a 4-year period. Of the 27 fetuses that were diagnosed before the 30th week of pregnancy, only one survived to term. Out of 27 fetuses, 25 were aborted; 21 of these 25 cases had severe hydrops. Spontaneous regression occurred in the second trimester in two of the 27 fetuses, both with Noonan syndrome stigmata. Seventeen fetuses were successfully karyotyped, including nine without abnormalities, seven with 45 X, and one with trisomy 21. The incidence of polyhydramnios (0 vs. 67%), extra anomalies (12 vs. 67%), and consanguinity or history of abnormal pregnancies (0 vs. 89%) was lower in fetuses with atypical karyotypes. Only three fetuses survived in the mentioned study^9^. On the other hand, in our study, approximately 20% of the cases had no composite adverse outcome, slightly higher than the mentioned research.

The main strengths of the present study were the determination of a cut-off value for NT in predicting composite adverse outcome, a relatively high number of study parameters, and the evaluation of middle-term outcomes. However, retrospective design, single-center experience, and lack of information related to the long-term outcomes were the main limitations.

In conclusion, CH cases with larger NT values had higher composite adverse outcome rates, and physicians should be more careful in the management of CH cases with NT≥5.2 mm values. Closer prenatal follow-up, comprehensive assessment for fetal structural abnormalities, and detailed antenatal counseling may be useful in the management of cases with NT values above this cut-off.

## ETHICS APPROVAL

This study was approved by the Local Ethics committee and the approval number is E2-23-5193.
